# BLOSSOM Dietary Habits and 1-Year Intravesical Recurrence in High-Risk Non-Muscle-Invasive Bladder Cancer Treated with BCG

**DOI:** 10.3390/curroncol33020128

**Published:** 2026-02-22

**Authors:** Carlo Buonerba, Raffaele Baio, Felice Crocetto, Dario Bruzzese, Francesco Del Giudice, Antonio Nacchia, Francesco Chiancone, Concetta Ingenito, Oriana Strianese, Antonio Verde, Ferdinando Costabile, Luca Scafuri, Roberto Sanseverino, Elena Sorrentino, Vittorio Riccio, Dalila Carino, Margherita Bertoni, Federica Monaco, Paolo Verze, Teresa Di Lauro, Sisto Perdonà, Celeste Manfredi, Antonio Ruffo, Gabriele Barbato, Serena Rizzano, Sara Rizzano, Armando Pisapia, Marina Pisapia, Rossella Di Trolio, Emanuela Sergianni, Giuseppe Romeo, Francesca Cappuccio, Gennaro Sosto, Giuseppe Di Lorenzo

**Affiliations:** 1 Oncology Unit, “Andrea Tortora” Hospital, ASL Salerno, 84016 Pagani, Italy; 2 Associazione O.R.A. ETS―Oncology Research Assistance, 84134 Salerno, Italy; 3 Department of Urology, Umberto I, Nocera Inferiore, 84014 Salerno, Italy; 4 Urology and Andrology Unit, Department of Neurosciences, Reproductive and Odontostomatological Sciences, University of Naples Federico II, 80131 Naples, Italy; 5 Department of Public Health, University of Naples Federico II, 80131 Naples, Italy; 6 Department of Maternal-Infant and Urological Sciences, Sapienza University of Rome, Policlinico Umberto I Hospital, 00161 Rome, Italy; 7 Sant’Andrea Hospital, 00189 Rome, Italy; 8 Department of Urology, A.O.R.N. Antonio Cardarelli Hospital, 80131 Naples, Italy; 9 Department of Agricultural, Environmental and Food Sciences (DiAAA), University of Molise, Via Francesco de Sanctis snc, 86100 Campobasso, Italy; 10 P.O. “Santissima Maria della Pietà” Hospital, 80026 Casoria, Italy; 11 Department of Anesthesia, San Paolo Hospital ASL Napoli 1 Centro, 80125 Naples, Italy; 12 Department of Medicine, Surgery and Dentistry, “Scuola Medica Salernitana”, University of Salerno, Baronissi, 84081 Salerno, Italy; 13 S.C. Oncologia Clinica Sperimentale Uro-Ginecologica, IRCCS Istituto Nazionale Tumori “Fondazione G. Pascale”, 80131 Naples, Italy; 14 Urology, Istituto Nazionale Tumori-IRCCS-Fondazione G. Pascale, 80131 Naples, Italy; 15 Unit of Urology, Department of Woman Child and General and Specialized Surgery, University of Campania “Luigi Vanvitelli”, 80138 Naples, Italy; 16 Dipartimento di Medicina e di Scienze della Salute “Vincenzo Tiberio”, 86100 Campobasso, Italy; 17 Department of Anesthesia and Intensive Care, “Anastasia Guerriero” Hospital, Caserta Local Health Authority, Marcianise, 81100 Caserta, Italy; 18 Fondazione “Peppino Scoppa”, Angri, 84012 Salerno, Italy; 19 Unit of Melanoma, Cancer Immunotherapy and Development Therapeutics, Istituto Nazionale Tumori Istituto di Ricovero e Cura a Carattere Scientifico Fondazione G. Pascale, 80131 Naples, Italy; 20 School of Medicine, Vita-Salute San Raffaele University, 20132 Milan, Italy; 21 General Directorate, ASL Salerno, 84134 Salerno, Italy; 22 School of Medicine, UniCamillus “Saint Camillus International” University of Health Sciences, 00131 Rome, Italy

**Keywords:** non-muscle-invasive bladder cancer, intravesical BCG, diet, 24 h dietary recall, FOODCONS, recurrence, Firth logistic regression, nutrient density, Mediterranean Adequacy Index

## Abstract

Evidence on modifiable post-diagnosis factors influencing outcomes after intravesical Bacillus Calmette–Guérin (BCG) therapy for high-risk non-muscle-invasive bladder cancer (NMIBC) is limited. In this exploratory, feasibility-focused prospective multicenter cohort (March 2023–November 2024), BCG-naïve patients completed repeated interviewer-administered 24 h dietary recalls; prespecified food groups, selected foods, and nutrients were screened for associations with 1-year intravesical recurrence using Firth’s penalized logistic regression adjusted a priori for age, sex, and total energy intake, with false discovery rate control within each exposure family. Forty-six patients were enrolled; 41 had evaluable recurrence status, including 8 recurrences (19.5%). Participants were predominantly overweight (mean body mass index (BMI) 28.4 kg/m^2^) and had low adherence to a Mediterranean dietary pattern (median Mediterranean Adequacy Index 2.25). No dietary exposure met the within-family false discovery rate threshold; the smallest q-value was 0.361. Nominal inverse associations were observed for leafy green vegetables (OR per 1 SD 0.385; 95% CI 0.101–0.972) and for energy-adjusted zinc (OR 0.280; 95% CI 0.069–0.802) and magnesium intakes (OR 0.260; 95% CI 0.045–0.872), but these did not remain significant after FDR adjustment. These exploratory signals warrant replication in larger, biomarker-informed cohorts incorporating dietary biomarkers and immune profiling during BCG. Given the limited sample size and low number of recurrence events, these findings are strictly hypothesis-generating and should not be interpreted as evidence of definitive protective or risk dietary factors.

## Simple Summary

Non-muscle-invasive bladder cancer can recur after standard intravesical Bacillus Calmette–Guérin (BCG) therapy, leading to repeated cystoscopies and procedures. Because urinary metabolites from foods may influence inflammation and immune responses, we prospectively recorded diets in BCG-naïve patients with high-risk disease. Using repeated interviewer-administered 24 h dietary recalls during the first year of BCG treatment and follow-up, we examined whether food groups, specific foods, or nutrients were linked to recurrence within 1 year. In this small cohort, higher intakes of leafy green vegetables and selected minerals showed nominal inverse associations with recurrence in exploratory analyses, but the findings require confirmation in larger studies. Because the cohort was small, the results are intended to inform hypotheses for future research rather than to provide definitive evidence for clinical practice. Many participants were overweight and had low adherence to a Mediterranean-style dietary pattern, highlighting a potential supportive-care gap.

## 1. Introduction

Bladder cancer remains a major global health burden, with more than 600,000 new diagnoses and over 200,000 deaths estimated worldwide in 2022, and it is among the most frequently diagnosed malignancies in men [1]. Approximately three-quarters of patients present with non-muscle-invasive bladder cancer (NMIBC), a clinically heterogeneous entity characterized by a persistent tendency to recur and, in high-risk subgroups, a clinically meaningful risk of progression to muscle-invasive disease that drives morbidity and long-term surveillance intensity [2]. Because recurrence mandates repeated cystoscopies, transurethral resections, and adjuvant intravesical treatments over many years, NMIBC contributes disproportionately to survivorship burden and healthcare resource utilization relative to its stage distribution [2]. Following transurethral resection, intravesical Bacillus Calmette–Guérin (BCG) remains the cornerstone adjuvant therapy for high-risk NMIBC, supported by decades of randomized evidence and contemporary guideline endorsement for induction and maintenance schedules [2]. Nevertheless, a substantial proportion of patients experience recurrence or fail to achieve durable disease control despite adequate BCG exposure, and the downstream consequences—repeat resections, intensified intravesical regimens, or consideration of radical cystectomy—create a pressing need for strategies that can improve outcomes without adding significant toxicity [3]. While tumor biology, smoking, and occupational exposures are well-established determinants of bladder cancer risk, comparatively little is known about modifiable post-diagnosis factors that might influence treatment response and recurrence trajectories in NMIBC. Diet is a particularly compelling candidate because the urothelium is chronically exposed to urinary metabolites derived from foods, beverages, and supplements, and because nutritional status can shape systemic inflammation and immune competence, mechanisms that are biologically relevant to BCG, whose antitumor activity relies on coordinated immune activation within the bladder microenvironment [4]. At the population level, the epidemiologic evidence linking specific dietary exposures to bladder cancer incidence remains limited and heterogeneous, reflecting variability in exposure definitions, residual confounding, and differences in study design, and few studies address outcomes after intravesical therapy. Even less evidence is available for survivorship and recurrence endpoints, where the clinical stakes are high, and the timing of exposure relative to intravesical therapy may be critical. Notably, emerging prospective data suggest that diet could plausibly modify NMIBC outcomes, but findings remain inconsistent: in one cohort, patterns characterized by higher intakes of processed meats, fried foods, and refined grains were associated with increased risks of recurrence and/or progression, whereas “healthier” patterns were not uniformly protective [5]. More recently, in the Be-Well prospective study, higher intake of raw cruciferous vegetables (and related isothiocyanate exposure) appeared to amplify the protective association of BCG with recurrence risk, providing a mechanistically coherent signal aligned with the immunomodulatory and phase II enzyme-inducing properties of these phytochemicals [4]. These observations are hypothesis-generating and underscore key methodological challenges: diet is multidimensional; single baseline measurements may not reflect intake during induction and maintenance phases; and common tools such as food-frequency questionnaires can be prone to systematic and random error, potentially attenuating true meaningful associations. Accordingly, a more granular, protocol-driven characterization of dietary intake over time—capturing foods, preparation methods, and supplement use—may be necessary to identify candidate exposures that warrant mechanistic study or dietary intervention trials in this setting.

Against this background, we conducted a prospective, multicenter observational cohort study (BLOSSOM) to evaluate dietary habits in a homogeneous population of BCG-naïve patients with high-risk NMIBC undergoing standard-of-care intravesical BCG, using repeated interviewer-administered 24 h dietary recalls across the first year of treatment and follow-up. This study was designed as an exploratory, hypothesis-generating analysis rather than to provide definitive effect estimates. Our aim was to assess feasibility and to identify candidate dietary signals (food groups, selected foods, and nutrients) that could inform supportive-care hypotheses and guide larger, biomarker-informed prospective cohorts and intervention studies.

## 2. Materials and Methods

### 2.1. Study Design and Setting

**Figure 1:**
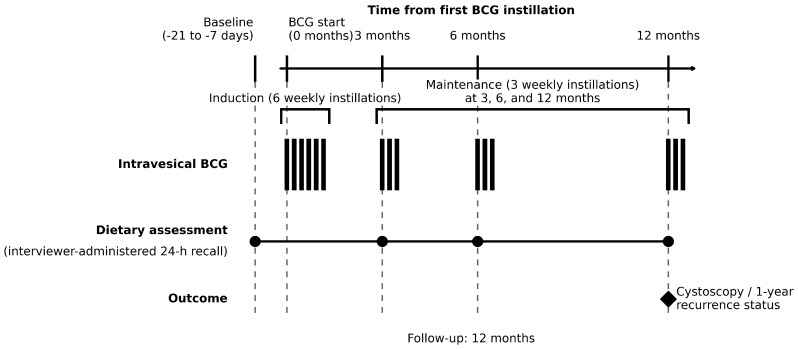
Study design and assessment schedule in BLOSSOM.

This study was designed as a prospective, observational cohort investigation of dietary habits in patients with high-risk non-muscle-invasive urothelial carcinoma of the bladder (NMIBC) initiating intravesical Bacillus Calmette–Guérin (BCG) therapy as part of standard clinical practice. The study was conducted across multiple Italian institutions with consecutive enrollment spanning March 2023 to November 2024. Recruitment was discontinued before reaching the planned target sample size because accrual was slower than anticipated for feasibility reasons (e.g., the time and staffing required for repeated interviewer-administered dietary recalls during BCG care). Enrollment was consecutive at each site and was not modified based on outcomes; there was no interim analysis that influenced the decision to stop recruitment. Participants were followed for 12 months from the first BCG instillation; protocol-scheduled evaluations were planned at baseline (approximately −21 to −7 days before the first BCG instillation) and at 3 ± 1, 6 ± 1, and 12 ± 1 months after the first BCG instillation, reflecting routine care pathways for high-risk NMIBC. The study timeline, including intravesical BCG administration, dietary recall time points, and 1-year outcome ascertainment, is summarized in Figure 1.

### 2.2. Patient Population and Procedures

Eligible participants were BCG-naïve adults with histologically proven high-risk NMIBC, diagnosed after the transurethral resection of bladder tumor (TURBT) and re-TURBT when clinically indicated, with no evidence of extravesical disease by standard staging procedures, and with planned intravesical BCG treatment; all participants provided written informed consent. Key protocol-specified exclusion criteria included muscle-invasive disease; low- or intermediate-risk NMIBC; recent radiotherapy and/or systemic antineoplastic treatment; ECOG performance status >2; contraindications to intravesical BCG; prior BCG administration; and life expectancy <1 year. Intravesical BCG was administered per routine practice, consisting of a 6-week induction course of weekly instillations followed by maintenance instillations given weekly for 3 weeks at 3, 6, and 12 months from therapy initiation. Dietary intake was assessed using repeated 24 h dietary recalls administered by trained interviewers using the Food Consumption Database (FOODCONS), a web-based platform developed by the Research Center for Food and Nutrition of the Council for Agricultural Research and Economics (CREA). Interviews followed a standardized multiple-pass approach consistent with EFSA EU Menu guidance for harmonized dietary data collection [6,7]. Dietary exposures were operationalized as the participant-specific mean across all available 24 h recalls; given the limited number of recalls per participant, this summary was intended primarily to rank individuals by intake rather than to precisely estimate absolute habitual intake. This approach is consistent with European survey guidance recommending two non-consecutive 24 h recalls for standardized dietary monitoring, and with validation work indicating that two standardized recalls can provide acceptable ranking performance for selected nutrients, while acknowledging that additional repeat measures improve precision [6,7,8,9,10]. Recalls were collected on non-consecutive days and were scheduled across different days of the week (weekday/weekend) when feasible to capture day-to-day variability; baseline assessment occurred before BCG induction, and follow-up assessments were aligned with routine visits during the first year (including planned maintenance time points). Total energy intake (kcal/day), food-group intakes, and nutrient intakes were derived from the FOODCONS output. Dietary variables were organized into three pre-defined exposure families: Group 1 (food groups), Group 2 (selected food items), and Group 3 (nutrients). In the locked dataset used for these analyses, at least one recall was available for each participant; the number of recall interviews per participant had a median of 2 (IQR, 1–3). Because the number of recurrence events was limited, we did not model dietary exposures as time-varying covariates (i.e., separate time-specific exposures at 3, 6, and 12 months) and instead used the cumulative-average exposure described above in all primary regression models. During interviews, participants were asked whether they were following a specific diet and whether they had made (or planned to make) meaningful dietary changes; no participant reported initiating a new dietary pattern during the study.

Baseline clinical variables available in the locked dataset included age, sex, pathologic stage indicators (pT1 and pTa), BCG induction completion, and completion of the 1-year BCG course. Detailed smoking history (current/former/never status, second-hand smoke exposure, and cumulative smoking intensity, such as pack-years) was not systematically captured in the locked dataset and therefore could not be included as a covariate in the regression models. The primary endpoint was 1-year intravesical recurrence (binary: 1 = yes, 0 = no). Participants with missing 1-year recurrence status were classified as lost to follow-up (LTFU) for the primary endpoint and were excluded from the regression analyses. The 1-year progression (binary) was captured as a secondary clinical endpoint and was defined as pathologically confirmed progression to muscle-invasive disease (≥pT2) and/or radiologic or histologic evidence of extravesical (regional or distant) disease during follow-up, as documented in the medical record. The five participants without 12-month outcome ascertainment were lost to follow-up before the 1-year time point and therefore did not have evaluable recurrence status at 12 months; we had no indication that outcome availability depended on recurrence itself, but informative missingness cannot be excluded.

### 2.3. Statistical Analysis

Sample size was pre-specified to provide preliminary evidence on associations between dietary exposures summarized as cumulative averages across available recalls and 1-year recurrence using logistic regression. Assuming an overall 1-year recurrence proportion of approximately 20% and considering an odds ratio (OR) of 0.50 per 1 SD increase in a dietary exposure as the smallest clinically meaningful effect, the protocol estimated that 103 participants would provide 80% power at a two-sided α = 0.05 for a univariate association; this was conservatively inflated to 148 to account for multivariable adjustment and collinearity (assumed R^2^ = 0.30; variance inflation factor = 1.43).

Baseline characteristics were summarized on the full, locked dataset using counts and percentages for categorical variables and mean (standard deviation [SD]), median (interquartile range [IQR]), and range for continuous variables.

Associations between each pre-defined dietary exposure and 1-year recurrence were evaluated using Firth’s penalized logistic regression to mitigate small-sample bias and to provide stable estimation under separation [11]. Models were fitted using the logistf package [12]. *p*-values and confidence intervals were derived using penalized likelihood ratio tests and profile penalized likelihood, respectively, as implemented in logistf [12]. Each dietary exposure was entered one at a time in a multivariable model adjusting a priori for age (years), sex, and total energy intake (kcal/day). Given the low number of recurrence events, covariate adjustment was intentionally restricted to minimize overfitting; consequently, additional important confounders (including smoking history) could not be adjusted for.

For continuous dietary exposures, total energy adjustment followed a multivariable nutrient density specification, expressing exposures as intake per 1000 kcal (X/energy_kcal × 1000) and additionally including total energy intake as a covariate to support interpretation as diet composition effects at a given energy intake [13]. Energy-adjusted continuous exposures were standardized (z-score) so that ORs reflect the relative change in the odds of 1-year recurrence for a 1 SD increase in the energy-adjusted exposure. Binary exposures, when applicable, were modeled as 1 versus 0.

The regression analysis set was restricted a priori to participants with non-missing values for recurrence status, age, sex, and total energy intake; observations with non-positive energy intake were excluded because energy density variables require division by energy. Within each exposure-specific model, participants were further restricted to complete cases for that exposure. For Group 2 selected food items, missing values (when present) were treated as non-consumption (set to 0). Where the number of consumers (intake > 0) was fewer than 10, the exposure was collapsed to any consumption versus none to improve model stability; in the locked dataset analyzed here, no Group 2 item met this threshold.

As an additional analysis requested during peer review, we fitted a post hoc, multivariable Firth penalized logistic regression model with 1-year recurrence as the dependent variable and age, sex, total energy intake, leafy green vegetables, zinc, magnesium, and potassium entered simultaneously. Dietary variables in this multivariable model were energy-adjusted using the same density specification (per 1000 kcal) and standardized (z-scores). Benjamini–Hochberg q-values were computed across the four dietary predictors only (leafy green vegetables, zinc, magnesium, and potassium).

To address multiplicity across the exploratory dietary exposure screen, *p*-values were adjusted within each pre-defined exposure family (food groups, selected food items, and nutrients) using the Benjamini–Hochberg procedure to control the false discovery rate at 0.05, and corresponding q-values were reported [14]. Analyses were performed using R and the readxl, dplyr, tidyr, and logistf packages (logistf version 1.26.1) [12,15].

## 3. Results

### 3.1. Patient Population and Baseline Characteristics

Between March 2023 and November 2024, a total of 46 consecutive, BCG-naïve patients with histologically confirmed high-risk non-muscle-invasive bladder cancer (NMIBC) were enrolled across the multiple participating institutions (A.O.R.N. Antonio Cardarelli Hospital, Naples, *n* = 6 [13.0%]; Sant’Andrea Hospital, Rome, *n* = 7 [15.2%]; University of Salerno “Scuola Medica Salernitana”, Salerno/Baronissi, *n* = 6 [13.0%]; University of Naples Federico II in cooperation with the University of Campania “Luigi Vanvitelli”, Naples, *n* = 6 [13.0%]; Sapienza University of Rome, Rome, *n* = 6 [13.0%]; and other collaborating sites aggregated within the ASL Salerno network, *n* = 15 [32.6%]) and completed at least one FOODCONS interviewer-administered 24 h dietary recall. Recruitment was discontinued before reaching the protocol target sample size due to slower-than-anticipated accrual and not due to outcome-guided enrollment decisions. One-year recurrence status was available for 41/46 participants (5/46 were lost to follow-up), and 8/41 experienced intravesical recurrence (center-level recurrence range, 14.3–20.0%). Baseline demographic, anthropometric, dietary, and clinical characteristics of the overall locked cohort are presented in Table 1.

The cohort was predominantly male (42/46, 91.3%), with a mean (SD) age of 65.5 (8.2) years and a median age of 67.0 years (IQR, 59.3–71.0; range, 45–79). Participants had a mean (SD) body weight of 84.6 (12.0) kg and height of 172.5 (7.0) cm, corresponding to a mean (SD) body mass index (BMI) of 28.4 (3.5) kg/m^2^ (median 27.5; IQR, 26.3–30.0; range, 23.2–40.4). Mean (SD) total reported daily energy intake was 1770.0 (450.6) kcal/day (median 1729.4; IQR, 1422.0–2101.6; range, 1017.8–2789.6). Adherence to a Mediterranean dietary pattern, summarized by the Mediterranean Adequacy Index (MAI), was poor, with a mean (SD) of 2.6 (1.2), and a median of 2.25 (IQR, 1.80–3.15; range, 0.7–5.9).

With respect to tumor characteristics at baseline, most participants had pathologic stage pT1 disease (33/46, 71.7%), while the remainder had pTa disease (13/46, 28.3%). Regarding intravesical therapy delivery, BCG induction was completed by 42/46 participants (91.3%). Completion of the planned 1-year BCG course was documented in 31/42 participants (73.8%) among those with available data, with 4 participants missing information for this variable.

### 3.2. Follow-Up and Outcomes

Outcome ascertainment at 1 year was available for the majority of the enrolled cohort. Specifically, 1-year intravesical recurrence status was available for 41/46 participants (89.1%), while 5/46 participants (10.9%) were classified as lost to follow-up (LTFU) before the 1-year time point for the primary recurrence endpoint. Among participants with evaluable 1-year follow-up (*n* = 41), intravesical recurrence within 1 year occurred in 8/41 participants (19.5%). These 41 participants comprised the evaluable population for the primary endpoint analyses. Under extreme assumptions for the 5 participants lost to follow-up, the 1-year recurrence proportion in the enrolled cohort would range from 17.4% (assuming none recurred) to 28.3% (assuming all recurred).

One-year progression status was available for the same 41 participants with recurrence ascertainment. Within this evaluable set, progression events were observed in 3/41 participants (7.3%) during the first year of follow-up.

### 3.3. Dietary Exposures and 1-Year Recurrence

In multivariable Firth’s penalized logistic regression models adjusted a priori for age, sex, and total energy intake, each dietary exposure (energy-adjusted intake per 1000 kcal, standardized to a z-score) was evaluated one at a time for association with 1-year recurrence among participants with available follow-up (*n* = 41; events = 8). Across the food-group (Group 1), selected food-item (Group 2), and nutrient (Group 3) exposure families, no associations met the within-family Benjamini–Hochberg false discovery rate threshold, with the smallest adjusted q-value equal to 0.361. In Group 2, higher intake of leafy green vegetables was inversely associated with recurrence in the nominal screen (OR per 1 SD increase: 0.385; 95% CI 0.101 to 0.972; *p* = 0.043), but this association did not remain significant after FDR adjustment (q = 0.361). In the nutrient family (Group 3), higher energy-adjusted zinc and magnesium intakes were inversely associated with recurrence (zinc: OR 0.280; 95% CI 0.069 to 0.802; *p* = 0.015; magnesium: OR 0.260; 95% CI 0.045 to 0.872; *p* = 0.025), but neither association remained significant after FDR adjustment (both q = 0.385). Detailed model results are reported in Table 2, Table 3 and Table 4. To aid interpretation of exposure stability for selected food items, Table 3 reports the number (%) of participants with non-zero consumption for each Group 2 item. In the additional multivariable model including leafy green vegetables, zinc, magnesium, and potassium simultaneously (adjusted for age, sex, and total energy intake), none of the dietary predictors remained statistically significant after Benjamini–Hochberg correction (all q > 0.05).

## 4. Discussion

This prospective, multicenter cohort of BCG-naïve patients with high-risk NMIBC evaluated whether dietary exposures captured by repeated interviewer-administered 24-hour recalls were associated with 1-year intravesical recurrence during standard-of-care BCG [2]. Recruitment stopped early, and the analysis is explicitly hypothesis-generating; nonetheless, the observed recurrence rate (~20% among patients with evaluable follow-up) illustrates the continuing clinical burden that drives repeated endoscopic procedures and escalation strategies despite guideline-concordant BCG [2,3,16]. In exploratory, energy-adjusted Firth models, higher zinc, magnesium, and potassium intakes and higher leafy green vegetable consumption were nominally associated with lower recurrence, but none of these signals remained significant after within-family false discovery rate adjustment [11,14]. Accordingly, BLOSSOM did not identify any dietary exposure as definitively protective or harmful with respect to 1-year intravesical recurrence; the reported associations should be interpreted as exploratory signals only.

A diet–BCG interaction is biologically plausible because BCG efficacy relies on coordinated innate and adaptive immune activation in the bladder, with downstream Th1-skewed pathways and cytotoxic effector activity considered important for tumor control [17,18]. Moreover, human data suggest that intravesical BCG can induce systemic trained-immunity signatures, implying durable innate reprogramming that could be sensitive to nutritional and metabolic context [19]. Within this framework, zinc is a compelling candidate for exposure. Zinc is essential for thymic function, T-cell signaling, and cytokine production; previous studies have shown that zinc status modulates IL-2 production and IL-2-dependent T-cell proliferation, and zinc deficiency impairs multiple immune parameters that are relevant to anti-tumor responses [20,21]. Accordingly, this nominal zinc association should be interpreted as hypothesis-generating; it motivates a testable hypothesis that zinc status (assessed with dietary and biomarker measures) may correlate with immune responsiveness and recurrence during BCG and should be examined in larger, adequately powered cohorts with immune readouts during induction and maintenance [18,19,21].

Host immune phenotype may also influence BCG responsiveness. For example, baseline absolute basophil count has been associated with shorter time to recurrence in BCG-treated high-grade T1 disease, suggesting that integrating readily available immune markers with nutritional and metabolic profiling could be informative in future, adequately powered cohorts [22].

The parallel association with leafy green vegetables may reflect a broader diet-quality construct. Prospective cohort meta-analysis evidence suggests that green leafy vegetable intake is inversely associated with bladder cancer risk, even when total fruit/vegetable findings are mixed [23]. In NMIBC survivorship, the Be-Well cohort reported that higher isothiocyanate exposure (linked to cruciferous vegetables) may modify recurrence risk in patients receiving intravesical therapy, providing a mechanistically coherent signal given urinary exposure to phytochemical metabolites and their immunomodulatory and redox effects [4]. Leafy greens also cluster with micronutrients (including magnesium and potassium) and with displacement of ultra-processed foods; therefore, future analyses should prioritize pattern-based modeling and substitution frameworks rather than single-nutrient interpretations, ideally complemented by objective exposure measures when feasible.

A clinically important finding in BLOSSOM is the lifestyle profile: participants were predominantly overweight (mean BMI ~28 kg/m^2^) and showed low Mediterranean pattern adherence (median MAI 2.25). Competing mortality is also clinically relevant in NMIBC survivorship: in a large multi-institutional BCG-treated high-grade T1 cohort, 150 all-cause deaths were observed, of which 77 were bladder cancer-related [22]. In population-based analyses, cardiovascular disease is the leading non-cancer cause of death after bladder cancer diagnosis, with diabetes mellitus also contributing meaningfully [24]. The MAI was designed to quantify proximity to a reference Italian-Mediterranean diet; historical Italian cohorts generally reported higher MAI values, suggesting substantial deviation in this NMIBC population [25]. Metabolic context may matter for intravesical immunotherapy: retrospective data link elevated BMI to higher risks of recurrence and progression among BCG-treated high-risk NMIBC patients [26,27]. Complementarily, a prospective analysis of the UroLife cohort found that better adherence to lifestyle recommendations after NMIBC diagnosis was associated with a lower risk of first recurrence, supporting the clinical logic of structured post-diagnosis counseling [28]. At the broader survivorship level, meta-analytic syntheses associate higher Mediterranean diet adherence with lower cancer mortality in the general population and lower all-cause mortality among cancer survivors, although certainty varies by endpoint [29]. Taken together, even if BLOSSOM cannot establish causality, it identifies a supportive-care gap that is potentially addressable with dietitian-led counseling integrated into NMIBC pathways, consistent with survivorship and clinical nutrition guidance [30,31].

Key limitations should temper interpretation. The small sample size and low event count yield imprecise estimates and increase the probability of chance findings, consistent with loss of significance after multiplicity correction [14]. Accordingly, BLOSSOM is underpowered to detect modest associations and cannot provide definitive effect estimates or support causal inference or clinical dietary recommendations. Dietary measurement was also limited by feasibility: the median of two recalls may not capture usual intake during the critical window of BCG induction/maintenance, and within-person variability can attenuate true associations. Accordingly, our dietary summary is expected to be more reliable for ranking individuals than for estimating absolute habitual intake, and any non-differential measurement error would tend to attenuate diet-recurrence associations toward the null [6,7,8,9,10]. Residual confounding is also plausible—particularly by smoking history (current, former, and second-hand exposure), diabetes/metabolic syndrome and related medications (e.g., glucose-lowering and lipid-lowering agents), and socioeconomic factors—which could not be adjusted for given the limited event count and incomplete capture of these variables in the locked dataset. Given the strong association of smoking with NMIBC outcomes, the lack of smoking adjustment could have materially influenced the direction and magnitude of the observed diet–recurrence associations. Although multiple-pass recalls are methodologically strong, misreporting remains a concern—particularly in individuals with overweight/obesity—and residual confounding is plausible because diet quality correlates with broader lifestyle and clinical factors that were not comprehensively captured [32]. Finally, BLOSSOM did not test whether dietary change during therapy modifies outcomes, an important question suggested by evidence that post-diagnosis lifestyle may be more prognostically relevant than pre-diagnosis behavior in NMIBC [28]. In addition, because exposures were summarized as participant-specific cumulative averages across available recalls, we were not able to evaluate diet as a time-varying covariate or to identify potentially critical exposure windows during BCG induction and maintenance. Finally, 5/46 participants (10.9%) were lost to follow-up before 12 months; although we had no indication that loss to follow-up depended on recurrence, informative missingness cannot be excluded.

Feasibility can be improved with a lower-burden assessment. The USDA Automated Multiple-Pass Method has been validated against doubly labeled water and remains a benchmark for interviewer-administered recalls, while web-based tools (e.g., ASA24) demonstrate acceptable validity relative to true intake measures and interviewer methods [32,33]. Recent validation studies further support web-based recalls and indicate that multiple administrations improve precision [34]. Beyond recalls, AI-enabled image-assisted dietary assessment may reduce respondent burden, but requires rigorous external validation, transparency, and professional oversight [35]. Emerging multimodal systems that combine vision models with retrieval from authoritative nutrient databases show promise for more scalable dietary phenotyping, and could support future NMIBC cohorts with higher measurement density and lower staff burden [36].

## 5. Conclusions

This exploratory, feasibility-focused prospective cohort indicates that high-risk NMIBC patients undergoing BCG frequently have overweight and low Mediterranean diet adherence, highlighting an opportunity for structured nutritional counseling within NMIBC care. In exploratory analyses, higher zinc intake and leafy green vegetable consumption were nominally inversely associated with 1-year recurrence; however, no association remained significant after within-family FDR control, and these findings should be interpreted cautiously as hypothesis-generating and requiring replication. This study highlights a potential supportive-care opportunity and identifies biologically plausible dietary signals that merit testing in larger, biomarker-informed cohorts incorporating dietary biomarkers and immune profiling during BCG. Overall, BLOSSOM should be interpreted as a feasibility and hypothesis-generating study, and larger, adequately powered cohorts are required before any definitive conclusions or clinical recommendations can be made.

## Figures and Tables

**Table 1:** Baseline characteristics of the overall cohort (N = 46).

Characteristic	N (%) or Mean (SD)	Median (IQR); Min–Max
Sex	Males: 42 (91.3%); Females: 4 (8.7%)	
Age, years	65.5 (8.2)	67.0 (59.3–71.0); 45–79
Total energy intake, kcal/day	1770.0 (450.6)	1729.4 (1422.0–2101.6); 1017.8–2789.6
Weight, kg	84.6 (12.0)	80.0 (76.3–94.8); 60–110
Height, cm	172.5 (7.0)	172.5 (170.0–178.0); 155–185
Body mass index, kg/m^2^	28.4 (3.5)	27.5 (26.3–30.0); 23.2–40.4
Mediterranean Adequacy Index (MAI)	2.6 (1.2)	2.25 (1.80–3.15); 0.7–5.9
Pathologic stage, pT1	33/46 (71.7%)	
Pathologic stage, pTa	13/46 (28.3%)	
BCG induction completed	42/46 (91.3%)	
Planned 1-year BCG course completed	31/42 (73.8%); data missing for 4 participants	
1-year recurrence status available	41/46 (89.1%); data missing for 5 participants	
1-year recurrence among those available	8/41 (19.5%)	
1-year progression status available	41/46 (89.1%); data missing for 5 participants	
1-year progression among those available	3/41 (7.3%)	

Note: SD, standard deviation; IQR, interquartile range; MAI, Mediterranean Adequacy Index; BCG, Bacillus Calmette–Guérin.

**Table 2:** Group 1 (food groups) and 1-year recurrence (*n* = 41; events = 8).

Exposure (Group 1: Food Groups)	OR	95% CI	*p*-Value	q-Value	N (Events)
Meat, processed meats, and meat substitutes	0.566	0.209 to 1.216	0.148	0.593	41 (8)
Cereals, bakery products, and cereal substitutes	2.035	0.854 to 6.358	0.116	0.593	41 (8)
Fruit, fresh and processed	0.510	0.183 to 1.167	0.115	0.593	41 (8)
Legumes, fresh and processed	0.609	0.012 to 1.255	0.212	0.635	41 (8)
Oils and fats	0.665	0.210 to 1.490	0.351	0.843	41 (8)
Milk, dairy products, and milk substitutes	0.805	0.336 to 1.684	0.576	0.863	41 (8)
Eggs	1.207	0.589 to 2.413	0.575	0.863	41 (8)
Vegetables, fresh and processed	1.295	0.561 to 2.979	0.534	0.863	41 (8)
Alcoholic beverages and substitutes	1.079	0.254 to 2.159	0.857	0.969	41 (8)
Sweets and substitutes	1.013	0.466 to 1.944	0.969	0.969	41 (8)
Potatoes, tubers, and products	0.933	0.277 to 2.256	0.890	0.969	41 (8)
Fish and seafood	1.091	0.469 to 2.361	0.828	0.969	41 (8)

Note: ORs are per 1 SD increase in the energy-adjusted exposure (density per 1000 kcal; z-scored), estimated by Firth’s penalized logistic regression adjusted for age, sex, and total energy intake. OR, odds ratio; CI, confidence interval; SD, standard deviation.

**Table 3:** Group 2 (selected food items) and 1-year recurrence (*n* = 41; events = 8).

Exposure (Group 2: Selected Food Items)	OR	95% CI	*p*-Value	q-Value	N (Events)	Consumers, n (%)
Extra-virgin olive oil and olive oil	0.533	0.149 to 1.306	0.191	0.361	41 (8)	41 (100.0%)
Coffee	1.974	0.869 to 5.652	0.107	0.361	41 (8)	41 (100.0%)
Raw tomatoes	0.622	0.220 to 1.382	0.258	0.361	41 (8)	23 (56.1%)
Cooked tomatoes	1.770	0.765 to 5.090	0.192	0.361	41 (8)	38 (92.7%)
Dark chocolate	1.612	0.655 to 3.486	0.233	0.361	41 (8)	11 (26.8%)
Leafy green vegetables	0.385	0.101 to 0.972	0.043	0.361	41 (8)	33 (80.5%)
Nuts	1.608	0.721 to 3.387	0.213	0.361	41 (8)	14 (34.1%)
Milk, yogurt, and cheese	0.803	0.333 to 1.682	0.572	0.728	41 (8)	40 (97.6%)
Tap water	1.129	0.521 to 2.205	0.730	0.852	41 (8)	21 (51.2%)
Cruciferous vegetables	0.934	0.427 to 1.884	0.847	0.913	41 (8)	18 (43.9%)
Cured meats (cold cuts)	0.982	0.469 to 1.785	0.953	0.953	41 (8)	31 (75.6%)
Citrus fruits	0.492	0.062 to 1.240	0.168	0.361	41 (8)	27 (65.9%)
Red meat	0.478	0.112 to 1.112	0.092	0.361	41 (8)	36 (87.8%)
Water	0.352	0.085 to 1.108	0.076	0.361	41 (8)	41 (100.0%)

Note: ORs are per 1 SD increase in the energy-adjusted exposure (density per 1000 kcal; z-scored), estimated by Firth’s penalized logistic regression adjusted for age, sex, and total energy intake. OR, odds ratio; CI, confidence interval; SD, standard deviation. Consumers are participants with non-zero intake (>0) for the selected item among those with evaluable 1-year recurrence status (*n* = 41).

**Table 4:** Group 3 (nutrients) and 1-year recurrence (*n* = 41; events = 8).

Exposure (Group 3: Nutrients)	OR	95% CI	*p*-Value	q-Value	N (Events)
Zinc	0.280	0.069 to 0.802	0.015	0.385	41 (8)
Magnesium	0.260	0.045 to 0.872	0.025	0.385	41 (8)
Total protein	0.513	0.132 to 1.132	0.104	0.445	41 (8)
Complex carbohydrates	1.921	0.855 to 5.251	0.121	0.445	41 (8)
Iron	0.442	0.097 to 1.199	0.129	0.445	41 (8)
Potassium	0.387	0.114 to 0.983	0.046	0.445	41 (8)
Phosphorus	0.559	0.215 to 1.180	0.129	0.445	41 (8)
Riboflavin (vitamin B2)	0.509	0.181 to 1.164	0.112	0.445	41 (8)
Vitamin B6	0.540	0.189 to 1.185	0.129	0.445	41 (8)
Vitamin C	0.523	0.145 to 1.224	0.148	0.460	41 (8)
Oleic acid	0.563	0.163 to 1.353	0.220	0.515	41 (8)
Monounsaturated fatty acids	0.591	0.183 to 1.391	0.249	0.515	41 (8)
Available carbohydrates	1.660	0.734 to 4.356	0.236	0.515	41 (8)
Thiamine (vitamin B1)	0.570	0.138 to 1.308	0.213	0.515	41 (8)
Niacin (vitamin B3)	0.620	0.230 to 1.340	0.230	0.515	41 (8)
Polyunsaturated fatty acids	1.455	0.639 to 3.036	0.330	0.639	41 (8)
Linoleic acid	1.384	0.600 to 2.860	0.400	0.688	41 (8)
Vitamin E	0.714	0.262 to 1.513	0.399	0.688	41 (8)
Linolenic acid	1.397	0.502 to 2.986	0.437	0.700	41 (8)
Beta-carotene	1.279	0.636 to 2.634	0.451	0.700	41 (8)
Fiber	0.738	0.241 to 1.738	0.510	0.752	41 (8)
Cholesterol	1.219	0.569 to 2.571	0.591	0.809	41 (8)
Soluble carbohydrates	0.824	0.327 to 1.771	0.630	0.809	41 (8)
Retinol	1.141	0.315 to 1.978	0.668	0.809	41 (8)
Vitamin K	0.844	0.324 to 1.780	0.675	0.809	41 (8)
Vitamin B12	0.842	0.272 to 1.764	0.679	0.809	41 (8)
Total fat	0.879	0.359 to 1.890	0.752	0.863	41 (8)
Saturated fatty acids	1.089	0.485 to 2.243	0.821	0.909	41 (8)
Calcium	1.047	0.432 to 2.323	0.913	0.943	41 (8)
Vitamin D	0.966	0.348 to 1.694	0.909	0.943	41 (8)
Alcohol	1.005	0.219 to 2.196	0.992	0.992	41 (8)

Note: ORs are per 1 SD increase in the energy-adjusted exposure (density per 1000 kcal; z-scored), estimated by Firth’s penalized logistic regression adjusted for age, sex, and total energy intake. q-values are Benjamini–Hochberg FDR-adjusted within the nutrient family. OR, odds ratio; CI, confidence interval; SD, standard deviation; FDR, false discovery rate.

## Data Availability

The data presented in this study are available on request from the corresponding author. The data are not publicly available due to privacy and ethical restrictions.

## Abbreviations

The following abbreviations are used in this manuscript:

**Abbreviation**	**Definition**
ASA24	Automated Self-Administered 24 h Dietary Assessment Tool
BCG	Bacillus Calmette–Guérin
Be-Well	Bladder cancer Epidemiology and Wellness study
BMI	Body mass index
CI	Confidence interval
CREA	Council for Agricultural Research and Economics
ECOG	Eastern Cooperative Oncology Group
EFSA	European Food Safety Authority
FDR	False discovery rate
FOODCONS	Food Consumption Database
IL	Interleukin
IQR	Interquartile range
LTFU	Lost to follow-up
MAI	Mediterranean Adequacy Index
NMIBC	Non-muscle-invasive bladder cancer
OR	Odds ratio
RCT	Randomized Controlled Trial
SD	Standard deviation
TURBT	Transurethral resection of bladder tumor
Th1	T helper type 1
Th2	T helper type 2
